# Neurofilament light chain may serve as a cross-species blood biomarker to assess aging and predict mortality

**DOI:** 10.1371/journal.pbio.3003606

**Published:** 2026-02-19

**Authors:** Carina Bergmann, Lisa M. Häsler, Marius Lambert, Stephan A. Kaeser, Stephanie A. Schultz, Barbara Riond, Marco Weiss, Martina Balz, Tobias Knauf-Witzens, Mathias Jucker

**Affiliations:** 1 German Center for Neurodegenerative Diseases (DZNE), Tübingen, Germany; 2 Department of Cellular Neurology, Hertie Institute for Clinical Brain Research, University of Tübingen, Tübingen, Germany; 3 Department of Neurology, Harvard Medical School, Boston, Massachusetts, United States of America; 4 Clinical Laboratory, Department of Clinical Diagnostics and Services, Vetsuisse Faculty, University of Zürich, Zürich, Switzerland; 5 LABOKLIN GmbH & Co. KG, Bad Kissingen, Germany; 6 WILHELMA Zoological-Botanical Gardens, Stuttgart, Germany; Stanford University, UNITED STATES OF AMERICA

## Abstract

Blood levels of neurofilament light chain (NfL) increase with age in healthy humans and have been shown to predict all-cause human mortality. To determine whether this relationship is conserved across species, we analyzed NfL in the blood of various animals. We observed age-related increases in NfL levels comparable to those seen in humans in mice, cats, dogs and horses. Longitudinal analysis of NfL trajectories in aged mice demonstrated that a faster rate of NfL increase predicts mortality. When comparing baseline NfL levels across 13 species, we found that those with lower baseline NfL levels tended to have longer lifespans; however, the collinearity between body size and life span complicates the interpretation of this finding. NfL was also robustly detected in blood of 39 additional mammalian species, as well as a few reptiles and birds, consistent with a conserved amino acid sequence of the NfL fragment in blood. Given the growing interest in NfL as a biomarker for neurological health and mortality in humans, our findings suggest that NfL may serve as a cross-species blood biomarker for assessing aging interventions and predicting mortality.

## Abstract 2

Blood levels of neurofilament light chain (NfL) increase with age in healthy humans and can predict all-cause human mortality, but is this true across species? This study shows that NfL age-related blood levels are comparable across mice, cats, dogs and horses, and may serve as a cross-species biomarker for assessing aging interventions and mortality prediction.

## Introduction

Neurofilament light chain (NfL) is expressed in neurons of the central and peripheral nervous system, and elevated levels have been associated with neuronal injury and neurodegenerative diseases [1]. However, NfL also increases with aging in people without known neurological disorders [2]. NfL levels were first associated with an increased risk of all-cause mortality in centenarians and non-centenarians [3] and have subsequently been proposed as a prognostic marker for all-cause mortality in population-based studies with younger cohorts (mean ages of 45–73 years) [4–7]. To investigate the generality of the age-related increase in NfL to other species, we examined NfL levels in the blood of a variety of animal species using the “Single Molecule Array” (Simoa) technology. Furthermore, we employed longitudinal blood sampling in aging mice to examine NfL predictability of death in a second species beyond those reported in humans.

## Results

Blood samples from a total of *n* = 862 animals of 57 species were obtained from a commercial and an academic veterinary diagnostic laboratory, a local zoo, and the Hertie Institute for Clinical Brain Research. Blood samples with known evidence of neural injury were excluded before measurement. In addition, NfL was measured in longitudinal blood samples (*n* = 157 observations across 44 mice) from aging mice housed at the Hertie Institute for Clinical Brain Research. For comparison, human blood NfL measurements from a recently published human age cohort (*n* = 438) were included in the present analysis (see Methods for details).

**Fig 1:**
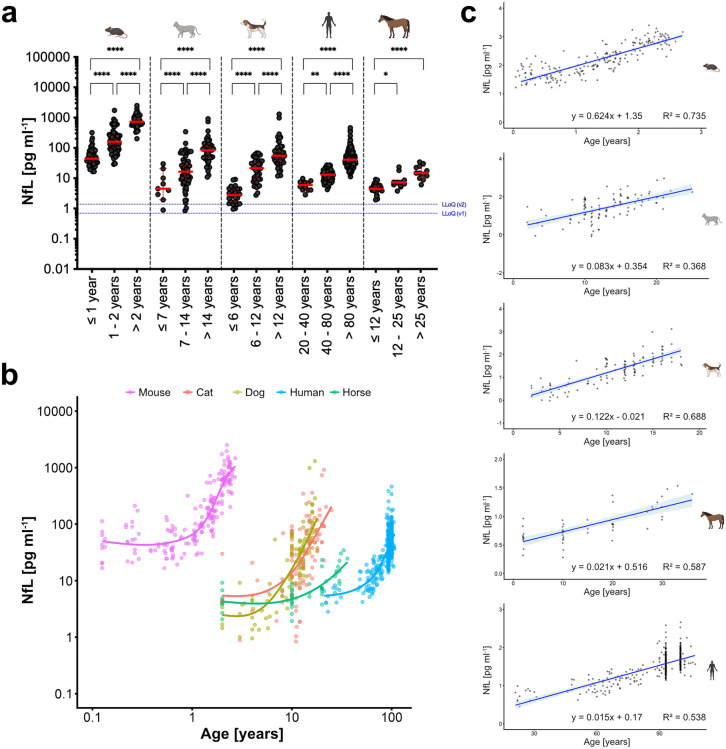
NfL levels in blood increase with age. **(a)** Mammalian species were grouped as young, adult, or aged depending on their known life expectancies. Shown are individual data points (y-axis (NfL [pg ml^−1^] on a log_10_ scale) with the median (red) and 95% confidence interval. For exact numbers per age group and species see S1 Table. Statistical comparisons were performed on log-transformed data to achieve normal distributions (which was achieved for all groups except adult horses and aged humans). Two-way ANOVA revealed a significant age effect (*F*(2,891) = 324.6, *p* < 0.0001), species effect (*F*(4,891) = 373.8; *p* < 0.0001) and an age × species interaction (*F*(8,891) = 6.962; *p* < 0.0001), the latter presumably based on the NfL increase being higher in cats, followed by mice and dogs, compared to humans and horses. The results of post-hoc Tukey’s multiple comparisons are shown (*p* < 0.05*; *p* < 0.001***; *p* < 0.0001****). Additional sensitivity analysis for horses (*H*(2)=28.75, *p* < 0.0001) and humans (*H*(2)=178.2, *p* < 0.0001) using Kruskal–Wallis test revealed significant differences among the age groups. Dunn’s post-hoc test for horses (young vs. adult: mean rank difference, −14.56, *p* = 0.0161; adult vs. aged: −12.67, *p* = 0.1219) and humans (young vs. aged: −244.1, *p* < 0.0001; adult vs. aged: −202.0, *p* < 0.0001). A separate ANOVA (type = 3) was performed including sex as an additional factor, but neither sex (*F*(1,573)=2.727; *p* = 0.099) nor any of the interactions with sex were significant (Species: *p* = 0.755; Age: *p* = 0.792). Note that the sex was not known for 17 animals and that it was not possible to assign the sex to 316 of the aged humans (see S1 Table). However, no effect of sex on NfL levels had been reported in this cohort of aged humans [3]. (**b**) Relationship between age [years] and NfL [pg ml^−1^] across the five species. Scatter points represent individual data, colored lines indicate generalized additive model (GAM) fits for each species (smoothing parameter *k* = 5, cubic regression spline basis). Both axes are shown on a log_10_ scale. (**c**) Age-dependent changes in NfL levels separated by species, with linear regression fit (log (NfL) ~ Age [years]) indicated by the blue line and the 95% confidence interval represented by the shaded area. *R*^2^ values indicate the proportion of variance in log(NfL) explained by age. Animal icons were created in BioRender (Bergmann, C. (2026) https://BioRender.com/6m960ov licensed under CC BY 4.0.). Illustrations were created with RStudio. Version(2025.09.1 + 401), GraphPad Prism10, and Affinity Designer (1.10.4). LLoQ = lower limit of quantification. The numerical data presented in this figure can be found in S1 Data (Fig 1). Note that human data have been censored due to data protection regulations.

To determine the relationship between NfL levels and age across species, we first categorized animal (mouse, cat, dog, horse) and human samples into three key life stages—young, adult, and aged—based on the known mean life span of the species. The threshold for the aged group was set at the mean life span itself, including all samples from individuals who had exceeded that age. The adult group was classified as those between half the mean life span and the mean life span. The young group comprised samples from individuals up to half of the mean life span (S1 Table). Samples from very young animals (first 5% of their mean life span, see Methods for details) were excluded due to reports from humans and calves that NfL is elevated in early postnatal stages [8,9]. Results revealed across all species age-related increases in NfL, with an observed 3-fold (horse), 7-fold (human), about 14-fold (mouse and dog), and 36-fold (cat) increase with age (Fig 1a). Sex differences (as far as this information was available, see S1 Table) were not significant.

To examine the age-related trajectories of NfL across species, baseline NfL measurements were modeled as a function of aging. In all four animal species, we observed non-linear associations and observed age trajectories similar to those in humans. Trajectories were characterized by a slow increase during adulthood and a sharp rise towards the end of life (Fig 1b and 1c).

**Fig 2:**
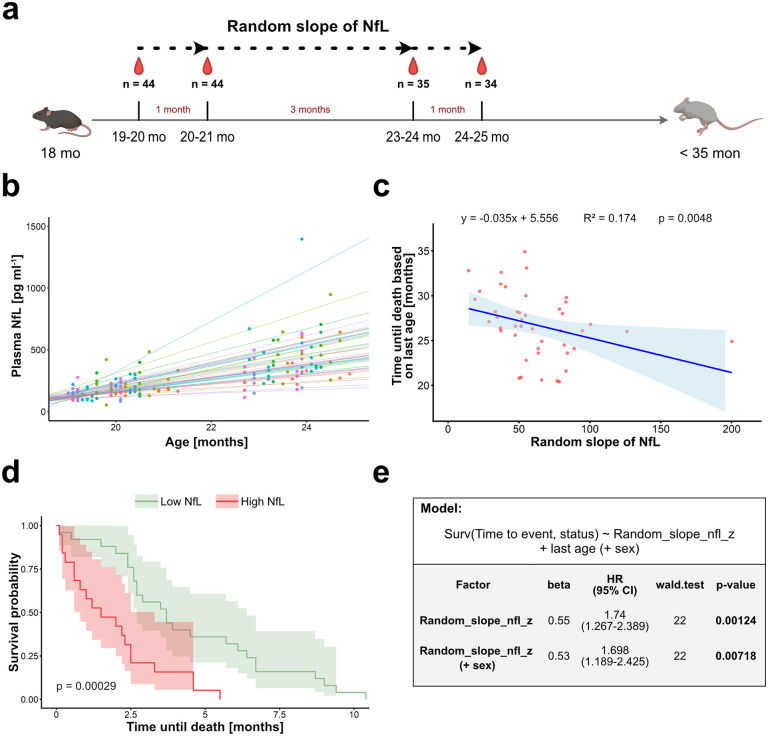
NfL trajectories in aged mice are associated with the time until death. **(a)** In aged C57BL/6J mice (initially *n* = 44 mice), blood plasma was collected retrobulbar longitudinally up to four times, with a recovery period of one or three-months in-between. Random slope of NfL (ΔNfL) refers to the individual slope of NfL with age for each animal, estimated from the random slope component of a linear mixed-effects model (NfL ~ Age + (1 + Age|Mouse)). (**b**) Scatter plot of plasma NfL [pg ml^−1^] versus age [months], color-coded by mouse, with mouse-specific regression line (random slope of NfL). (**c**) Time-to-death-dependent random slope of NfL, with linear regression fit (time until death ~ random slope of NfL) indicated by the blue line and 95% confidence interval represented by the shaded area. *R*^2^ indicates the proportion of variance in time until death explained by random slope of NfL, with *p* = 0.0048. (**d**) Kaplan–Meier survival curves with random slope of NfL above (red) or below (green) the mean of 62.587 [pg ml^−1^ month^−1^], reveal significant differences between the two curves. (**e**) Random slope of NfL was z-standardized (mean = 0, SD = 1) using the scale function in *R*. Subsequently, Cox proportional hazard regression analysis confirms a strong association between NfL change and increased mortality per standard deviation, independent of sex. The *p*-values were adjusted for multiple testing using Bonferroni correction (*n* = 2). Animal icons were created in BioRender (Bergmann, C. (2026) https://BioRender.com/6m960ov licensed under CC BY 4.0.). Illustrations were created with RStudio. Version(2025.09.1 + 401) and Affinity Designer (1.10.4). The numerical data presented in this figure can be found in S1 Data (Fig 2).

While longitudinal blood measurements and tracking of the animals until death were not possible for most species, we followed an independent cohort of C57BL/6J mice under laboratory conditions. This allowed us to assess within-individual changes in NfL as a function of aging. Blood was collected at 19−20 months of age and again one month later. This procedure was repeated three months later (at 23−24 months) and once more one month later, yielding a total of four longitudinal blood samples (Fig 2a; see Methods for details). The rate of change in NfL concentration (slope; ΔNfL) was calculated from a linear mixed-effects model (see Methods for details) and was inversely correlated with remaining life span (*p* = 0.0048; Fig 2b and 2c). Dividing the mice into two groups based on whether their NfL slope was higher or lower than the mean (62.587 pg·ml^−1^·month^−1^) and using Kaplan–Meier survival analysis, revealed a robust separation between the groups with mice with a lower slope surviving longer than mice with a higher slope (Fig 2d). Subsequent Cox regression analyses indicated that each standard deviation increase in NfL slope was associated with a 73.5% (or 69.1% including sex) higher mortality risk (Fig 2e).

**Fig 3:**
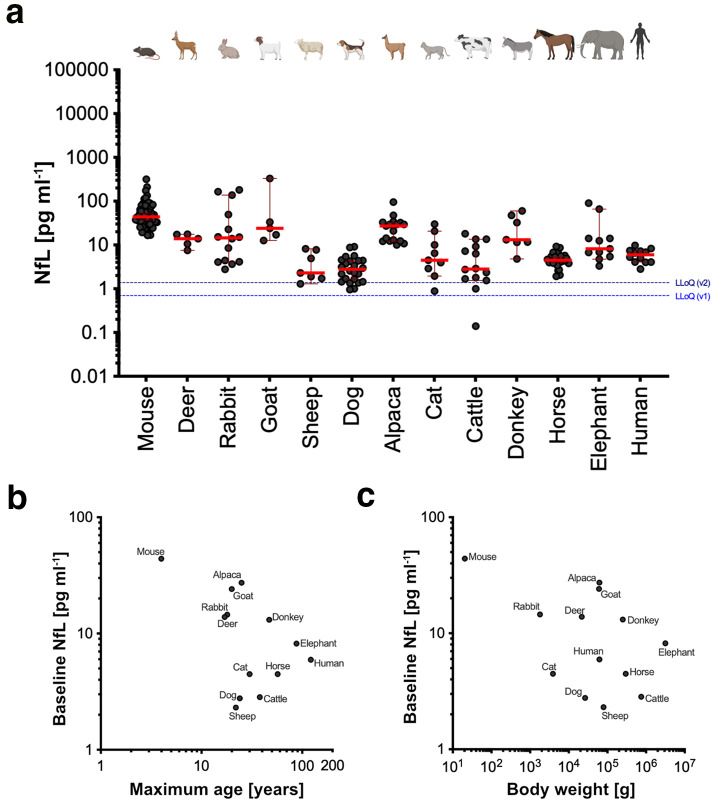
Baseline NfL levels in different species and their correlation with life span and body weight. (**a**) Baseline blood NfL levels, determined as the median NfL concentration within the young age group, differed among the 13 species analyzed (Kruskal–Wallis *H*(12) = 152.5, *p* < 0.0001). For exact animal numbers, see S2 Table. Shown are blood NfL levels (y-axis (NfL [pg ml^−1^] on a log_10_ scale) with the median (red) and 95% confidence interval. Illustrations were created with Biorender.com. (**b, c**) Nonparametric Spearman correlation analysis between (**b**) baseline NfL levels [pg ml^−1^] and maximum age [years] (*r* = −0.4286 [−0.7992 − 0.1781], *p* = 0.1460) and (**c**) baseline NfL levels [pg ml^−1^] and body weight [g] (*r* = −0.4670 [−0.8159 − 0.1311], *p* = 0.1103). Axes are shown on a log_10_ scale. LLoQ = lower limit of quantification. Animal icons were created in BioRender (Bergmann, C. (2026) https://BioRender.com/6m960ov licensed under CC BY 4.0.). Illustrations were created with GraphPad Prism10 and Affinity Designer (1.10.4). The numerical data presented in this figure can be found in S1 Data (Fig 3). Note that human data have been censored due to data protection regulations.

Next, we extended our analyses of NfL to a larger set of species. While blood samples from aged animals were relatively rare, NfL measurements for young animals with blood from at least 5 animals were available for 12 species in addition to humans (Fig 3a; S2 Table). Robust differences in baseline NfL levels were found between the species, with long-lived species tending to have lower levels (Fig 3a), although this association was not significant (Fig 3b).

Since body weight has been shown to be associated with NfL levels in humans [10], NfL levels were compared to the mean body weight within species. Results revealed again an inverse relationship between baseline NfL levels and body weight (Fig 3c). To account for differences in body weight in the correlation analysis between NfL levels and life span, we tested two models according to a previous publication [11]: NfL/body weight ~ maximum life span (*r* = −0.7253, *p* = 0.0067**) and NfL ~ maximum life span + body weight (*r* = −0.467, *p* = 0.1103). However, the high correlation between body weight and life span in the present dataset (*r* = 0.7637, *p* = 0.0034**) likely explains the strong correlation in the first model.

**Fig 4:**
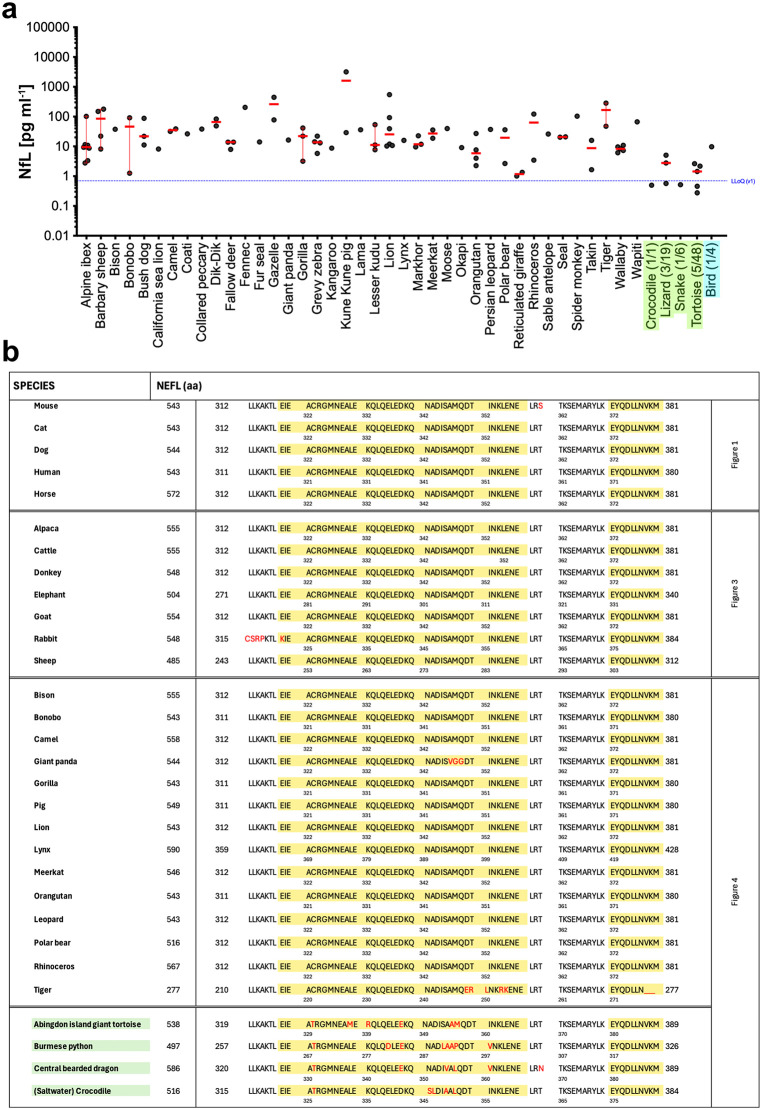
Amino acid sequence homology of the presumed blood NEFL fragment among species. (**a**) NfL [pg ml^−1^] on a log_10_ scale, showing the median (red) and 95% confidence intervals for various species. These species were not included in Figs 1–3 because only 1–5 blood samples/species were available, and the age of the donor animals was often unknown. (**b**) NEFL sequence that is detected by NfL antibodies UD1 and UD2 (yellow) (https://www.uniprot.org/uniprotkb?query=NEFL) used in the Simoa assay (NF-light) and in the present study [12,14] for mammals and reptiles (green) as close as possible to the species used in the present study. Red letters indicate amino acid substitutions compared to the human NEFL sequence. LLoQ = lower limit of quantification. Illustrations were created with GraphPad Prism10, Microsoft Excel (16.103.4), and Affinity Designer (1.10.4). The numerical data presented in this figure can be found in S1 Data (Fig 4).

Blood NfL could be measured in a further 39 mammalian species (Fig 4a). Consistently, the amino acid sequence of the NfL epitope critical for the present immunoassay [12–14] revealed a very high degree of conservation for all species for which the NfL sequence was publicly available (Fig 4b). While NfL could be detected in every mammalian blood sample examined, NfL could only be detected in 5 out of 48 (5/48) tortoises or turtles (Testudinidae, Geoemydidae, and Emydidae), in 1/6 snakes (royal python, boa constrictor, corn snake), in 1/1 crocodile, in 3/19 lizards (iguana, bearded dragon, scorpion bearded lizard), and in 1/4 birds (i.e., in a 29-year-old Amazon parrot, but not in any chicken or cockatiel. The reptiles and bird measurements may be false positives, but it is equally likely that healthy reptiles and birds have NfL levels below the detection limit of the current immunoassay.

## Discussion

We found significant age-related increases in NfL for all species for which blood samples were available across the entire age range. Age-related increases have previously been noted for dogs [15,16] and among adult cattle [8]. Increased levels of phosphorylated neurofilament heavy chain have been reported in horses [17]. Our data also suggest that NfL trajectories in aged mice predict mortality, as has been reported in aged humans [3–7].

Due to the nature of samples sent to veterinary diagnostic laboratories, many blood samples from this source are likely to come from animals with unknown health problems, which in turn may affect NfL levels. However, unknown health problems are expected to occur equally in samples from all age groups sent to a diagnostic laboratory. Moreover, for the analysis of aging and baseline NfL, the median was used to smooth out differences coming from individual health problems.

In humans, the age-related increase in NfL predicts the progression of neurodegenerative diseases [18] but is also associated with all-cause mortality in aging cohorts [3–7]. Consistently, large blood proteomic studies have identified NfL as one of the top proteins predictive for/associated with mortality [19,20], suggesting a link between mortality and the aging nervous system. While it has been suggested that underlying subclinical neurodegenerative diseases might drive the association between NfL levels and mortality in humans, neurodegenerative diseases as known in humans are rare in animals. While in aged cats, dogs and horses, aspects of Alzheimer pathology (Aβ plaques or Tau tangles) have been reported, importantly, C57BL/6J mice (as used in the present study) do not exhibit any signs or core pathological features of human neurodegenerative diseases [21]. This suggests that a faster NfL rate of change predicts death independently of underlying subclinical neurodegenerative diseases. A limitation of the experimental longitudinal mouse cohort design used here, with the final blood sample taken around 24 months of age, is that mortality was assessed in ‘aged’ mice, preventing conclusions whether changes in NfL at younger age would also be predictive of mortality. Additionally, the statistical basis for the mortality analysis rests on the critical assumption that the timing of the pre-defined humane endpoint is an accurate and unbiased proxy for the timing of natural death.

Variation in baseline NfL levels in the blood of mammals showed an inverse relationship with body size and life span, but the collinearity between these two factors makes the interpretation difficult. Differences in baseline NfL levels among species may reflect the brain/body mass ratio - larger brains have more NfL, and brain-derived NfL is more diluted in larger bodies. However, despite similarities in brain-to-body mass ratios between mice and humans [22], we observed a 7.4-fold difference in baseline (median) NfL concentration. NfL is also a component of the peripheral nervous system [1,23] and its scaling relative to body size is unclear.

An alternative explanation could be that baseline NfL levels align more closely with species-specific metabolic differences [24,25]. Smaller animals generally have higher metabolic rates and more rapid neuronal turnover, which may contribute to increased calpain-mediated neuronal NfL release [13]. This interpretation aligns with our previous finding that diet-restricted animals have lower NfL levels [3], which is consistent with reduced metabolic rates in diet-restricted animals and humans [26,27]. This could also explain why NfL levels are detectable in only a few reptile species, despite overall homology in the blood NfL fragment sequence. One possibility to address such mechanistic questions would be to assess NfL levels in species (e.g., naked mole rats, bats) that deviate from the typical body weight-life span correlation observed in mammals [11].

The sequence conservation of the blood NfL fragment among species suggests an evolutionarily conserved process of neuronal NfL release into the blood. For mice, a calpain-mediated release of NfL has been suggested with an immune (microglia) activation of the secreted NfL fragments [13] and microglia activation with aging is commonly observed in many species [28,29]. A conserved NfL-mediated immune mechanism is an exciting possibility and would advance NfL as a cross-species biomarker of neural aging [30].

The current findings open the door to broader applications of NfL measurements beyond human medicine. They demonstrate the feasibility of using NfL as a biomarker to track neurological health and assess potential aging interventions in non-human species with potential applications in veterinary medicine.

## Methods

### Animals and blood samples

Animal blood samples were obtained from a veterinary diagnostic laboratory (LABOKLIN, Bad Kissingen, Germany), from the Department of Clinical Diagnostics and Services of the University of Zürich (Zürich, Switzerland) and from the biobank of a local zoo (WILHELMA, Stuttgart, Germany). The vast majority of these samples were blood sera (>97%), while the rest were blood plasma samples. Samples were shipped to Tübingen on either dry or wet ice. All wet ice samples were stored at 4 °C and analyzed within 24–48 hours upon arrival. Previous research has found that human serum NfL remains stable at least up to 7 days at room temperature and withstands at least 3 freeze-thaw cycles [31,32]. Mouse plasma samples were obtained from the mouse biobank at the Hertie Institute for Clinical Brain Research (University of Tübingen, Germany). Some of the mouse samples with NfL measurements were included in a previous analysis [3]. NfL values of human plasma samples (except for one) were already included in a previous publication [3] and are included in the current analysis for comparison. Detailed numbers of animal and human samples are given in S1 and S2 Tables. The use of human plasma samples was approved by the ethics committee of the medical faculty of the University of Tübingen, Germany (project number 718/2014BO2). The collection of mouse plasma was in accordance with the regulations of the Veterinary Office of Baden-Württemberg, Germany, and was approved by the local animal care and use committees.

### Longitudinal blood collection and life-long monitoring of aging mice

For longitudinal blood sampling (up to four times with a recovery period of one- or three-months in-between) in aging C57BL/6J mice (*n* = 44), a maximum of 10% of the total blood volume was collected per time point, based on an approximate blood volume of 1.7 ml blood/ 25 g body weight [33]. Mice were anesthetized with 1.0%–1.5% isoflurane (induction with 5%) in a Link7 Anaesthesia & Evacuation All-in-One System (Patterson Scientific, Waukesha, WI). By puncturing the retro-bulbar sinus with a microhematocrit capillary (32 mm, 9 µl) blood was collected into a MiniCollect EDTA-coated tube. Once enough blood was collected, the capillary was removed, and clean dry gauze was applied to achieve haemostasis. The blood was centrifuged (10 min, 2000*g*, 4 °C) to obtain blood plasma, which was snap-frozen on dry ice and stored at −80 °C until further use.

After the blood collections, mice were observed multiple times per week until natural death (*n* = 4) or until their humane endpoint was reached (*n* = 40). The humane endpoint had been determined prior to the start of the study and was expected to reflect a point in time shortly before natural death. Criteria included physical appearance (coat, skin, eyes, nose, teeth), posture and activity (both undisturbed in the cage and in motion), nutritional status (feces/urine output, body weight, body condition (analogous to [34]), but also clinical and experiment-specific signs (respiratory rate, behavior after handling, wound healing disorders, unsuccessful treatment of injured/infected wounds) were assessed and evaluated with scores from 0 (normal) to 3 (significantly altered). As soon as a score of 8 or more was reached animals were euthanised.

### NfL measurements

Plasma and serum samples stored at −80 °C were thawed on wet ice for one hour. They were then mixed for 30 s and centrifuged at 10,000*g* for 5 min at 4 °C. Measurements were performed on a single molecule array platform (Simoa, HD-X analyzer; Quanterix) using commercially available assay kits (NF-Light Advantage Kit Cat 103186; *n* = 1,108 samples; NF-Light v2 Advantage Kit Cat 104073; *n* = 192 samples). Samples were automatically diluted 1:4 with Simoa NfL Sample Diluent. All samples were run in duplicate, and the mean was taken. Based on internal human cerebrospinal fluid control samples used in the Simoa NfL assays, results measured using the NF-Light v2 Advantage Kit were multiplied with a factor of 1.44, in order to match NfL results from NF-Light Advantage Kit. Coefficients of variation (CVs) in all samples were < 20% except for 13 samples (5× CV > 20%–25%; 8x > 25%–47%). In 28 samples, only one of the duplicates was successfully measured and one sample was excluded due to a CV of 70.7%. Samples were assayed blinded. LLoQ represents the lower limit of quantification (v1: NF-Light Advantage Kit (0.696 pg ml^−1^) and v2:NF-Light v2 Advantage Kit (1.38 pg ml^−1^)).

### Statistical analyses

#### Exclusion of data.

Samples from very young animals (first 5% of their mean life span, in total *n* = 24), were excluded due to reports from humans and calves that NfL is elevated in early postnatal stages [8,9]. In addition, a batch of 1-year-old cats (*n* = 20) and dogs (*n* = 16) were excluded because their exact age was uncertain (all with an age specified as exactly one year) and it was therefore unclear whether they fell within the first 5%. In fact, the inclusion of these 1-year-old cats and dogs skewed the generalized additive models (GAM) regression upwards to the left and the exclusion of these data ensured that the regression line reflects the age-related variations rather than early developmental effects (Fig 1b). A total of 34 samples were excluded (in Figs 1 and 3) due to missing age data, and a further 51 samples in Fig 3 were excluded because they exceeded the age range of the respective species and were therefore no longer considered young. No other data points were excluded.

#### Conversion of data.

Due to conflicting literature findings on NfL concentrations in plasma and serum samples, with 10%−20% higher values in serum [31,35], <10% higher values in plasma [36] or nearly equivalent values [37–39], NfL concentrations from plasma were treated similarly to NfL concentrations from serum in a 1:1 ratio. Absolute NfL levels of mice, cats, dogs, humans, and horses were not normally distributed. Therefore, log_10_-transformation was done prior to the aging analyses as indicated in the legend of Fig 1.

#### Longitudinal analysis of blood NfL and mortality prediction.

To determine the average monthly within-individual rate of change in NfL (individual slope estimates), a linear mixed-effects model (lme4, REML, Nelder-Mead optimizer) was fitted with time-varying NfL as the dependent variable, time-varying age as a fixed effect, and random intercepts and slopes per mouse (NfL ~ Age + (1 + Age|Mouse)). Individual slope estimates were extracted for downstream analyses. Time until death was calculated as the time between the age of the last NfL measurement available per mouse and the individual’s age at death. A linear regression fit (Time until death ~ ΔNfL) was applied to examine time until death as a function of ΔNfL (represented by the linear regression fit in blue and the 95% confidence interval). The equation and *R*^2^ indicate the proportion of variance in time until death explained by ΔNfL. For Kaplan–Meier survival estimates, cohort was stratified based on their ΔNfL being higher or lower than the cohorts mean. For multivariate Cox proportional hazard regression analysis, ΔNfL was z-standardized (mean = 0, SD = 1), using the scale function, before fitting of each predictor using the coxph function (survival package). For each model, regression coefficients, hazard ratios with 95% confidence intervals, Wald test statistics, and Bonferroni-adjusted *p*-values were extracted and summarized in a tabular form. Models that failed to converge were excluded.

#### Other statistical aspects.

Models, statistics, and calculations were conducted using RStudio (Version 2025.09.1 + 401), Prism 10 (GraphPad) or Microsoft Excel v.16. For Fig 1, the test for outliers was performed on log_10_-transformed data (Robust regression and outlier removal—ROUT with *Q* = 0.1% in Prism 10), whereby no data point was identified as such. For Figs 2, 3, and 4, we deliberately refrained from testing for potential outliers, as most datasets are very small, and apparent outliers may simply reflect true biological variability. Their removal would carry the risk of bias, which we wanted to avoid. Statistical significance was set at *p* < 0.05 and adjusted for multiple testing (including two-way ANOVA and post-hoc Tukey’s multiple testing, one-way ANOVA and Kruskal–Wallis test, as well as for the Cox proportional hazard regression analysis). For visual interpretation, GAM with cubic splines (*k* = 5) were used in Fig 1, plotting NfL as a function of age for each of the five species. Linear regression fits (log (NfL) ~ Age), separated by species, were applied to examine age-related changes in NfL (represented by the linear regression fit in blue and the 95% confidence interval). The equation specifies the relationship and *R*^2^ indicate the proportion of variance in log(NfL) explained by age. The nonparametric Spearman correlation was used for correlation analyses between baseline NfL levels in young animals and body weight or maximum age. All values (in Figs 1a, 3a, and 4a) are expressed as median ± 95% confidence interval. For detailed information, see figure legends.

## Supporting information

S1 Table Characteristics of humans and non-human species in which age-related changes in NfL were assessed. (TIFF)

S2 Table Characteristics of humans and non-human species in which baseline NfL levels were assessed. (TIFF)

S1 Data Raw data set, separated according to the corresponding figures. (XLSX)

## Abbreviations

CVs: coefficients of variation
GAM: , generalized additive models
NfL: neurofilament light chain
