# Supplementary figures and images for: Neurofilament light chain may serve as a cross-species blood biomarker to assess aging and predict mortality
Source: PLoS Biol. 2026 Feb 19;24(2):e3003606. doi: 10.1371/journal.pbio.3003606 (PMC12919815; doi:10.1371/journal.pbio.3003606)

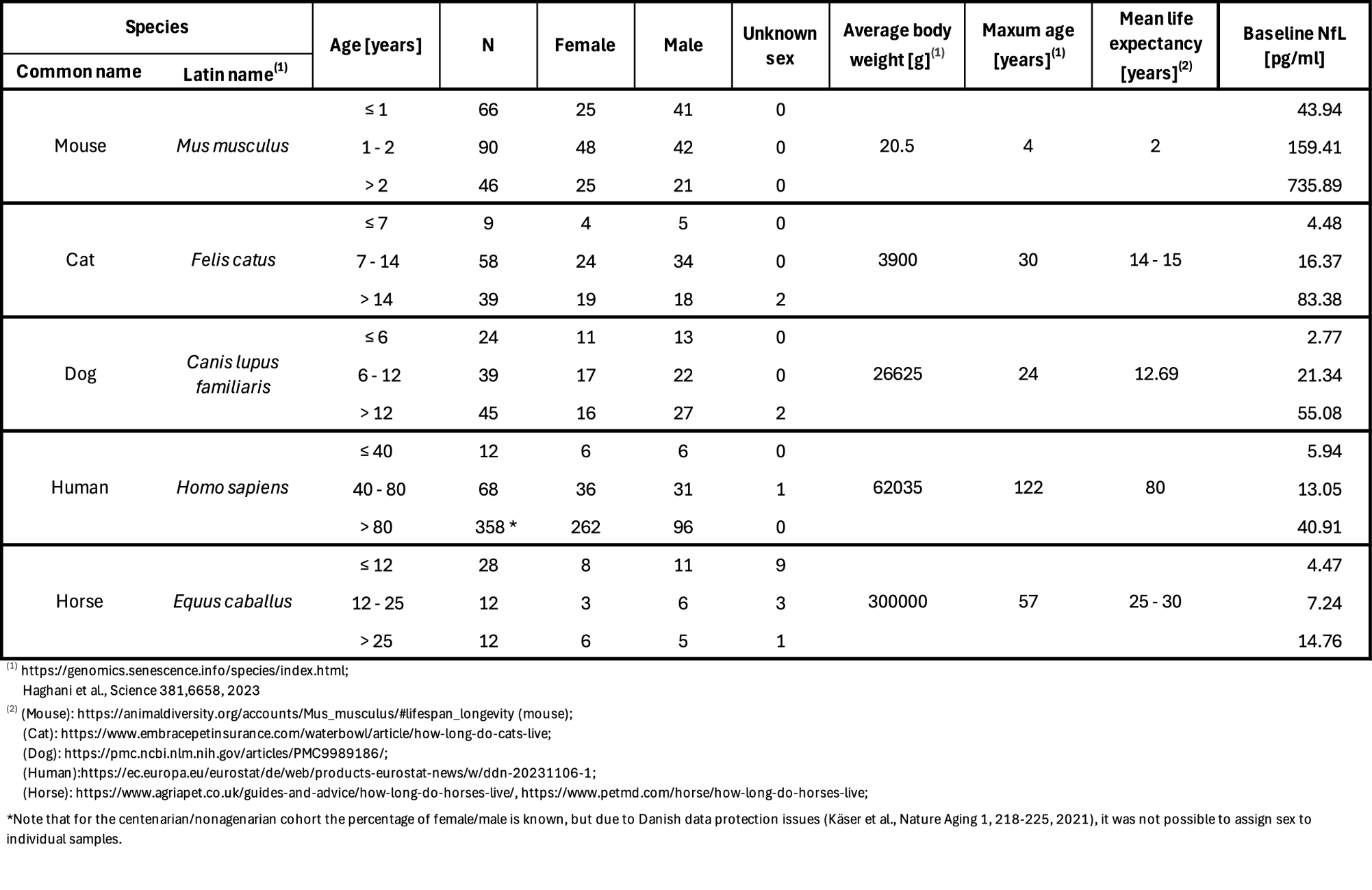

Supplement: S1 Table — (TIFF) [file pbio.3003606.s001.tiff]

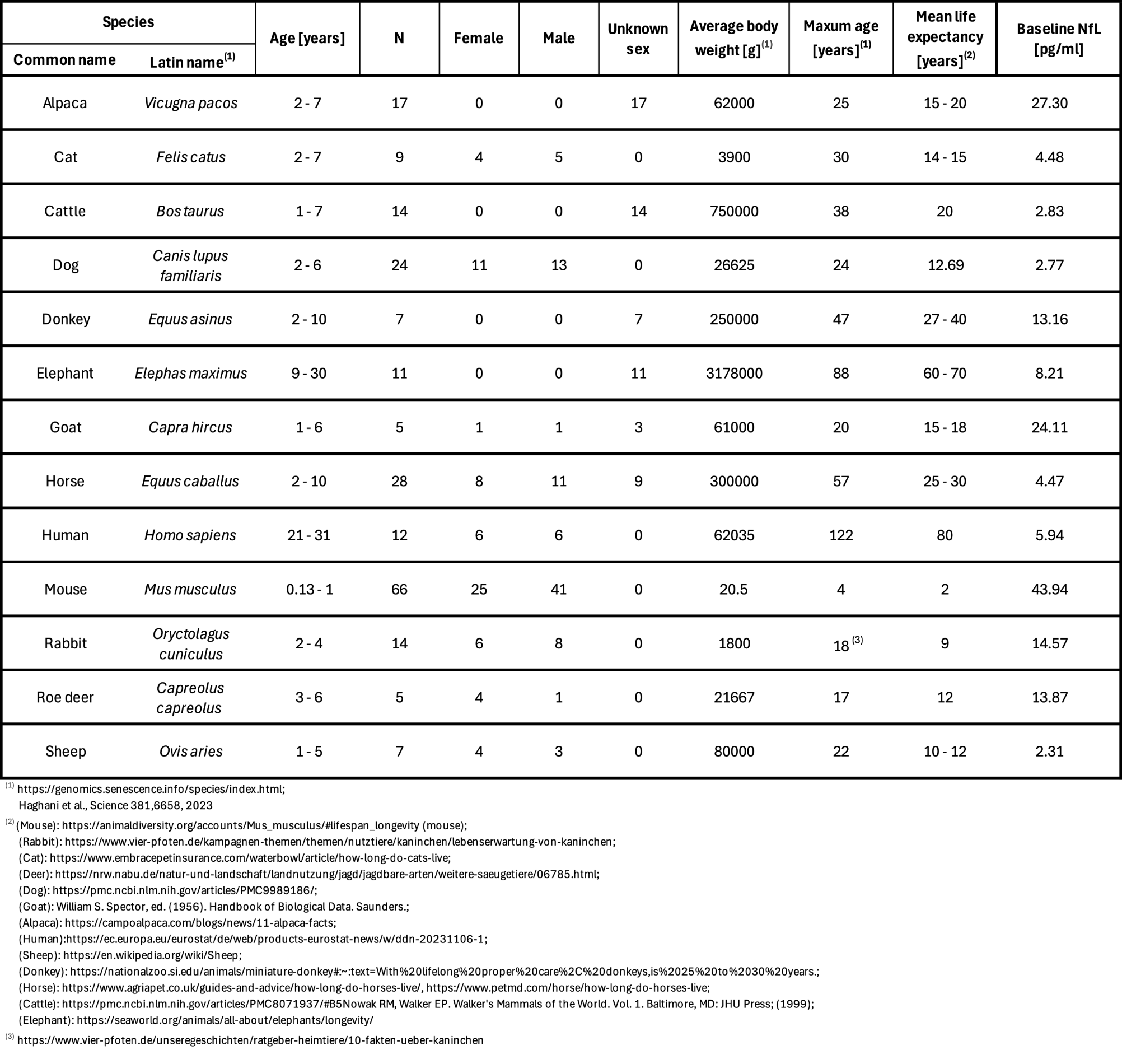

Supplement: S2 Table — (TIFF) [file pbio.3003606.s002.tiff]
